# Quality of Blastocysts Created by Embryo Splitting: A Time-Lapse Monitoring and Chromosomal Aneuploidy Study 

**DOI:** 10.22074/cellj.2020.6717

**Published:** 2019-12-15

**Authors:** Marjan Omidi, Mohammad Ali Khalili, Iman Halvaei, Fatemeh Montazeri, Seyed Mehdi Kalantar

**Affiliations:** 1 Research and Clinical Center for Infertility, Yazd Reproductive Sciences Institute, Shahid Sadoughi University of Medical Sciences, Yazd, Iran; 2 Department of Reproductive Biology, Shahid Sadoughi University of Medical Sciences, Yazd, Iran; 3 Department of Anatomical Sciences, Faculty of Medical Sciences, Tarbiat Modares University, Tehran, Iran; 4 Abortion Research Center, Yazd Institute of Reproductive Sciences, Shahid Sadoughi University of Medical Sciences, Yazd, Iran

**Keywords:** Aneuploidy, Blastocyst, Mosaicism, Time-Lapse

## Abstract

**Objective:**

The aim of this study was to screen the potential of human embryos to develop into expanding blastocysts following *in vitro* embryo splitting and then assess the quality of the generated blastocysts based on chromosomal characteristics and using morphokinetics.

**Materials and Methods:**

In this experimental study, a total of 82 good quality cleavage-stage donated embryos (8- 14 cells) were used (24 embryos were cultured to the blastocyst stage as controls and 58 embryos underwent *in vitro* splitting). After *in vitro* splitting, the blastomere donor and blastomere recipient embryos were named twin A and twin B, respectively. Morphokinetics and morphological parameters were evaluated using a time-lapse system in the blastocysts developed from twin embryos. Aneuploidy of chromosomes 13, 15, 16, 18, 21, 22, X and Y were analyzed in the twin blastocysts.

**Results:**

Following *in vitro* splitting, of the 116 resulting twin embryos, 80 (69%) developed to the expanded blastocyst (EBL) stage compared to 21 (87.5%) embryos in the control group (P>0.05). The morphokinetics analysis suggested that the developmental time-points were influenced by the *in vitro* splitting. Moreover, the blastocysts developed from A and B twins had impaired morphology compared to controls. Regarding chromosome abnormalities, there was no significant difference in the rate of aneuploidy or mosaicism between the different groups.

**Conclusion:**

This study showed that while no chromosomal abnormalities were seen, *in vitro* embryo splitting may affect the embryo morphokinetics.

## Introduction

Identical twins resulting from natural splitting of human embryos are accepted by society which are comparable with non-identical twins. Successful pregnancies following *in vitro* embryo splitting have been established in large animals, including sheep (1), cattle (2), horses (3) and pigs (4). The first attempt at *in vitro* human embryo splitting was carried out by Hall et al. (5) in 1993. In their study, the polyploid cleaved embryos underwent *in vitro* splitting and grew to the 32-cell stage. Later, efforts on *in vitro* human embryo splitting resulted in blastocysts which were morphologically suitable for clinical usage such as for "low responders" (6-8).

Successful pregnancy and live birth of healthy animals as well as morphologically normal adequate human blastocysts following *in vitro* embryo splitting increased the possibility of applying this method to infertile couples. However, application of *in vitro* splitting in the clinic requires comprehensive validation of the derived twin embryos. Up to now, the majority of studies have investigated the developmental competence of twin embryos after *in vitro* splitting and the data regarding cellular and molecular assessments in these embryos are very limited (6-9). Recently, Noli and colleagues showed that the majority of the cells in the twin blastocysts expressed inner cell mass (ICM) and trophectoderm (TE) markers simultaneously (8). Later, the same group evaluated the effects of *in vitro* embryo splitting on the miRNA profile of their spent blastocyst medium (SBM). They found the SBM from twin embryos had a significant difference in the amount of miRNAs involved in implantation compared to euploid implanted blastocysts (10). Generally, despite the possible advantages of this method for infertile patients, there is controversy over its clinical use in published studies (11). In addition, the chromosomal state of developed blastocysts from *in vitro* splitting has not been evaluated yet. Time-lapse monitoring (TLM), as a novel technology can be useful for embryo quality assessments through the evaluation of embryo morphology and developmental kinetics (12). The main goal in this study was to analyze the chromosomal status combined with developmental competence using TLM in human twin embryos created via *in vitro* splitting.

## Materials and Methods

The embryos were donated without any financial incentive. Informed consent was obtained from each couple. The Ethical Committee of our institute approved this experimental study since the embryos would not be transferred to the uterus after experimental procedures (IR.SSU.MEDICINE.REC.1395.93).

### Embryos

All day-2 or day-3 embryos were cryopreserved from 2011 to 2016 by vitrification using RapidVit™ Cleave kit (Vitrolife, Sweden). Donated embryos were warmed using RapidWarm™ Cleave kit (Vitrolife, Sweden) according to the manufacturer’s instructions. The warmed embryos were cultured *in vitro* until development to at least the 8-cell stage. The inclusive embryos with symmetrical blastomeres and no fragmentation or <10% fragmentation were considered as good quality embryos (13).

### Embryo micromanipulation and time-lapse monitoring

The good quality 8-14-cell embryos were preincubated in 5 µL microdroplets of Ca-Mg-free culture medium (PGD medium, Vitrolife, Sweden) prior to biopsy and covered with mineral oil for 3 minutes at 37˚C in order to facilitate the separation of blastomeres. A 1480 nm infrared diode laser (OCTAX Laser Shot®, MTG, Germany) was used to open a 35- 40 μm diameter hole in the zona pellucida (ZP). Half of the blastomeres were taken out using a micropipette with a 30 μm inner diameter (Sunlight Medical, Jacksonville, FL, USA) regardless of the presence or absence of the nucleus. The biopsied blastomeres were then inserted one by one from donor embryos (twin A) into a previously prepared empty ZP to create the recipient embryos (twin B). In this study, the empty ZPs were derived from immature oocytes or discarded embryos (14). After *in vitro* splitting, both twin A and twin B embryos were carefully washed and cultured individually in nine-micro well primo vision plates (Vitrolife, Sweden) which were prepared with 40 µL of G-2™ PLUS media (Vitrolife, Sweden) overlaid with mineral oil and equilibrated overnight in a triple-gas incubator. Images were acquired in seven distinct focal planes every 10 minutes by a primovision time-lapse system (Vitrolife, Sweden). Intact embryos without manipulation (controls) were cultured and developed under the same conditions. Time-lapse images by the primovision system were used for the assessment of embryo development, timing of developmental events, blastocyst morphology and morphometry.

### Morphokinetics analysis

**Fig 1:**
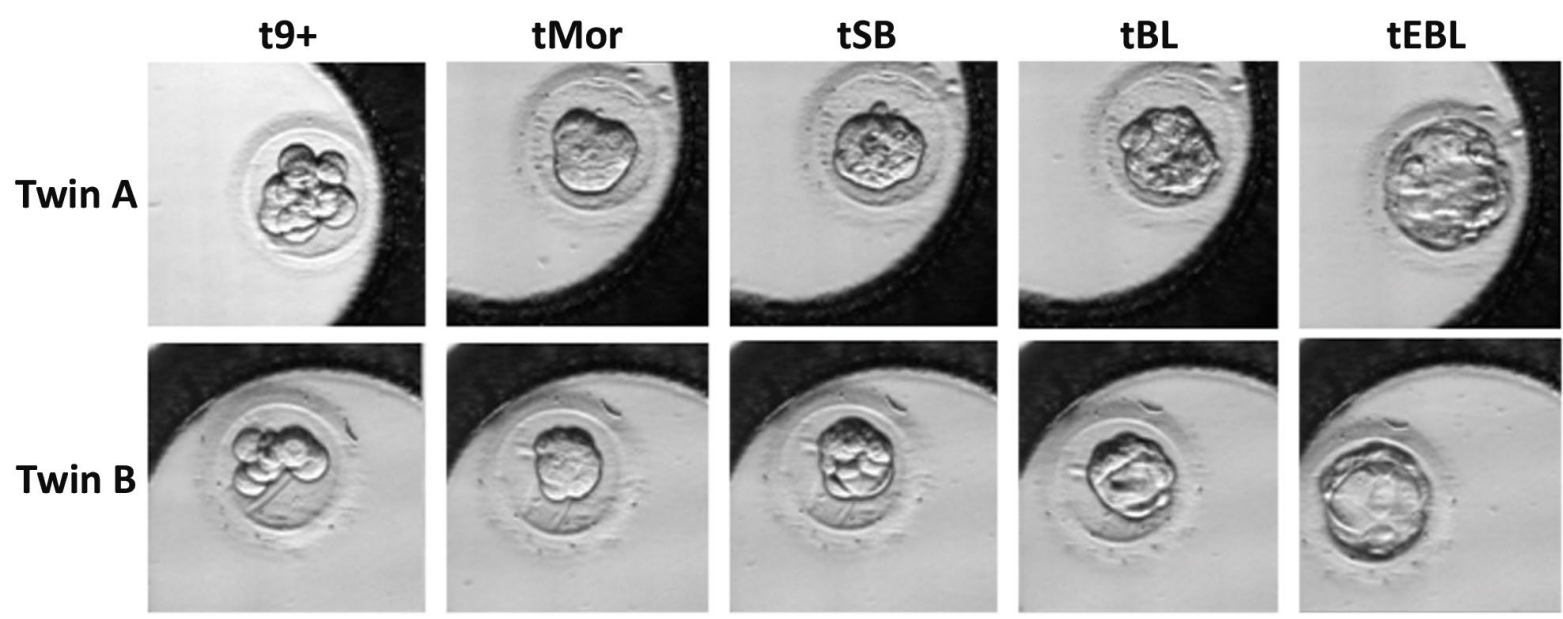
Developmental stages used for morphokinetic analyses using time-lapse monitoring compared between twin A and twin B. 9+; More than nine blastomeres, Mor; Morula or fully compacted embryo, SB; Start of blastulation, BL; Blastocyst, and EBL; Expanded blastocyst.

The developmental stages after *in vitro* splitting used for morphokinetics parameters were: the existence of more than nine blastomeres (9+), formation of the morula or fully compacted embryo (Mor), the start of blastulation (SB), formation of the blastocyst (BL) and formation of the expanded blastocyst (EBL) (Fig .1). The duration of stages was calculated as follows: compaction (9+ to Mor), start of blastulation (Mor to SB), blastocyst formation (SB to BL) and blastocyst expansion (BL to EBL).

### Morphology analysis

Blastocyst morphology was assessed using the images acquired from the time-lapse system. At the blastocyst stage, embryo quality was assessed based on Gardner’s classification, which takes into account the expansion grade and the development of the ICM and TE (15). According to this classification, we defined three blastocyst quality classes for full and expanded blastocysts: A) good- (AA, AB, BA and BB), fair- (AC, CA, BC and CB) and poor-quality blastocysts (CC).

### Morphometric analysis

The diameter (in micrometers) of the expanded blastocysts was measured by EmbryoViewer. The measurements were taken on the images of the blastocysts. The diameter of each blastocyst was calculated as the average of the distance between the outside borders of the TE measured in two directions (vertical and horizontal).

### Cytogenetic screening procedures Trophectoderm biopsy

Embryo biopsies were performed on a pre-warmed stage in a dish prepared with 5 µL droplets of HEPES buffered medium (G-MOPS, Vitrolife, Sweden) overlaid with pre-equilibrated mineral oil. The herniated TE cells were biopsied in the expanded blastocysts developed from A and B twins, through the previously created hole in the ZP. In the control embryos, a 10-20 µm hole was made in the ZP directly opposite the ICM of the blastocysts using a diode laser. Blastocysts were incubated for a further 4 hours to allow blastocoel expansion and herniation of the TE cells. After herniation, 5-10 TE cells were drawn into the biopsy pipette followed by laser-assisted cutting of the target cells.

### Fixation

The biopsied TE cells were washed in a hypotonic solution (6 mg/mL bovine serum albumin in 0.1% sodium citrate), then placed in a hypotonic solution for 3 minutes. The TE cells were then placed on a prewashed (with 100% ethanol) microscope slide. After that, an aliquot of fixative (methanol: acetic acid, 3:1) was dropped onto the specimen. Air was then blown across the sample to evaporate the fixative (16).

### Fluorescence *in situ* hybridization

The biopsied TE cells were fixed on glass slides as previously described (17). FISH assays of the fixed TE cells took place using two sequential hybridizations. The first hybridization contained probes for chromosomes 13, 18, 21, and X (MetaSystems, Altlussheim, Germany) and the second round was performed using probes for chromosomes 15, 16, 22, and Y (MetaSystems, Altlussheim, Germany). The prepared slides were examined under a fluorescence microscope (Olympus BX51, GSL-10 with BX61, Japan). Classification of embryos after FISH assay results was done according to the criteria published by f Delhanty et al. (18). In this classification, the embryos were categorized into four groups: normal, abnormal non-mosaic, diploid mosaic, and abnormal mosaic.

### Statistical analysis

Statistical analysis was performed using SPSS (SPSS version 20, Chicago, IL) and/or GraphPadPrism (GraphPad Software, San Diego, CA, USA). The quantitative and qualitative data were presented as mean ± SD and percentages, respectively. The Shapiro-Wilk test was applied to evaluate the normal distribution of data. t test was used for independent samples and one-way ANOVA (followed by Tukey’s test) as parametric and Mann-Whitney U and Kruskal-Wallis as nonparametric were used tests wherever appropriate. The chi-squared test was applied for comparison between qualitative data. P<0.05 was considered as significant.

## Results

### Developmental potential to expanding blastocyst is unaffected following embryo splitting

After warming, there were 82 good quality cleavagestage embryos. Among these, 58 embryos were split into two groups: group 1 (n=37), including embryos with 8- 9 blastomeres; and group 2 (n=21), including embryos with 10-14 blastomeres. The remaining 24 embryos in the same condition were used as the controls. In general, from 116 resulting twin embryos, 80 (69%) of them were developed to the EBL stage compared to 21 (87.5%) embryos in the control group. Moreover, developmental potential of A and B twins was similar regardless of their groups (70.7% vs. 67.2%, P= 0.688). Furthermore, when comparing twin and control embryos, the number of starting blastomeres appeared to have no significant effect on them reaching each stage.

Next, we compared the developmental potential of the embryos of different origins i.e. control, twin A or twin B. Although overall more embryos in the group 2 were developed to each stage compared to group 1, the only significant difference was in the number of embryos reaching the SB stage between twin B embryos: 73% of embryos in group 1 versus 95.2% of embryos in group 2 (P= 0.038).

### Dynamic pattern of twin embryos

**Fig 2:**
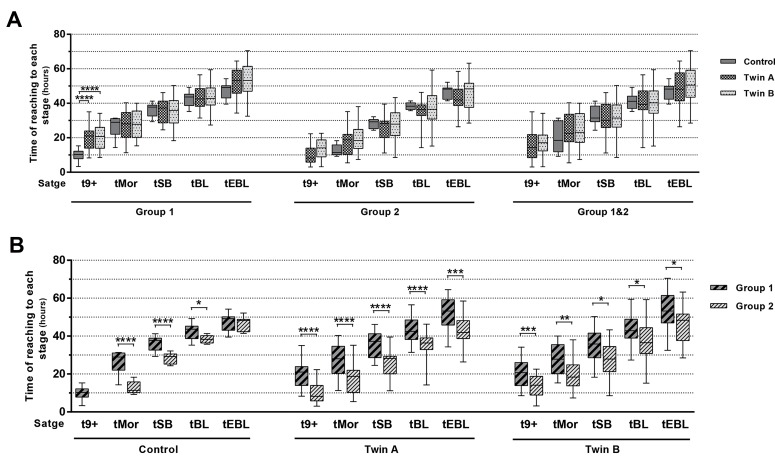
Developmental dynamics of twin embryos. **A.** The time of reaching each developmental stage for twin and control embryos within the group (group 1, 8-9 blastomeres and group 2, 10-14 blastomeres). **B.** Comparison of the time of reaching each developmental stage depending on the number of starting blastomeres in the control, twin A (donor blastomere) and twin B (recipient blastomere) embryos, separately. 9+; More than nine blastomeres, Mor; Morula or fully compacted embryo, SB; Start of blastulation, BL; Blastocyst, EBL; Expanded blastocyst, *; P≤0.05, **; P≤0.01, ***; P≤0.001, and ****; P≤0.0001.

Assessment and comparison of the developmental dynamics between twin and control embryos that reached the EBL stage was done regarding two parameters; time of reaching each stage and the duration between the stages. In comparing the time of reaching each stage, there was no significant difference between the control and twin embryos, except for time of reaching more than 9 blastomeres (t9+) in the group 1 (Fig .2A). The time these embryos took to get to this stage was significantly lower in the control embryos (9.80 ± 3.51 hours) compared to twins (twin A: 19.70 ± 7.05 hours and twin B: 20.54 ± 7.03 hours, P˂0.0001). In a different way, regarding the origin, the differences between the embryos in groups 1 and 2 were significant for the time the embryos took to reach all developmental stages (Fig .2B).

**Fig 3:**
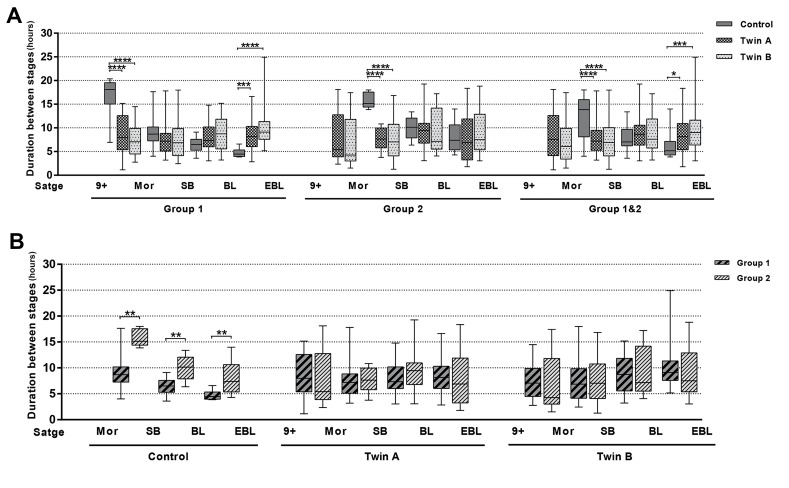
Developmental dynamics of twin embryos.** A.** Comparison of the duration between stages for twin and control embryos within the group (group 1, 8-9 blastomeres and group 2, 10-14 blastomeres). **B.** Comparison of the duration between stages depending on the number of starting blastomeres in the control, twin A (donor blastomere) and twin B (recipient blastomere) embryos, separately. 9+; More than nine blastomeres, Mor; Morula or fully compacted embryo, SB; Start of blastulation, BL; Blastocyst, EBL; Expanded blastocyst, *; P≤0.05, **; P≤0.01, ***; P≤0.001, and ****; P≤0.0001.

Comparison of twins and control embryos did not reveal a pronounced rhythm in their developmental dynamics regards to the duration of critical stages in embryo development. Although some significant differences were found between twin and control embryos at the compaction and expansion stages (Fig .3A). A and B twins belonging to groups 1 and 2, did not differ in duration between the different stages (Fig .3B).

### Blastocyst morphology and inner cell mass quality following splitting

The findings showed that the proportion of blastocysts with good morphology was significantly higher in the control group (71.4%) compared to A twins (39.6%, P=0.015) and B (28.6%, P=0.001). Although, the rate of fair quality embryos increased in the twins (A: 39.6% and B: 40.5%) after the splitting procedure compared to the control group (23.8%, Table 1). Furthermore, the sub-group analysis displayed an increased rate of grade C ICM and grade B TE in twin embryos (Table 1). Two (4.2%) ICMs in the twin A group were grade A. However, no grade A ICMs were noticed in the B twins.

### Decreased size of blastocysts developed from twin embryos

Morphometric analysis showed a significant decrease in the overall size of twin expanded blastocysts compared to controls (mean ± SD (µm): 102.35 ± 5.19 vs. 120.92 ± 4.55, P˂0.0001). Regardless of the number of starting blastomeres, the average diameter of blastocysts in A and B twins was 103.53 µm and 101.11 µm, respectively, whereas the average diameter for control embryos was 120.92 µm.

### No significant difference in the prevalence of aneuploidy or mosaicism in twin embryos

**Fig 4:**
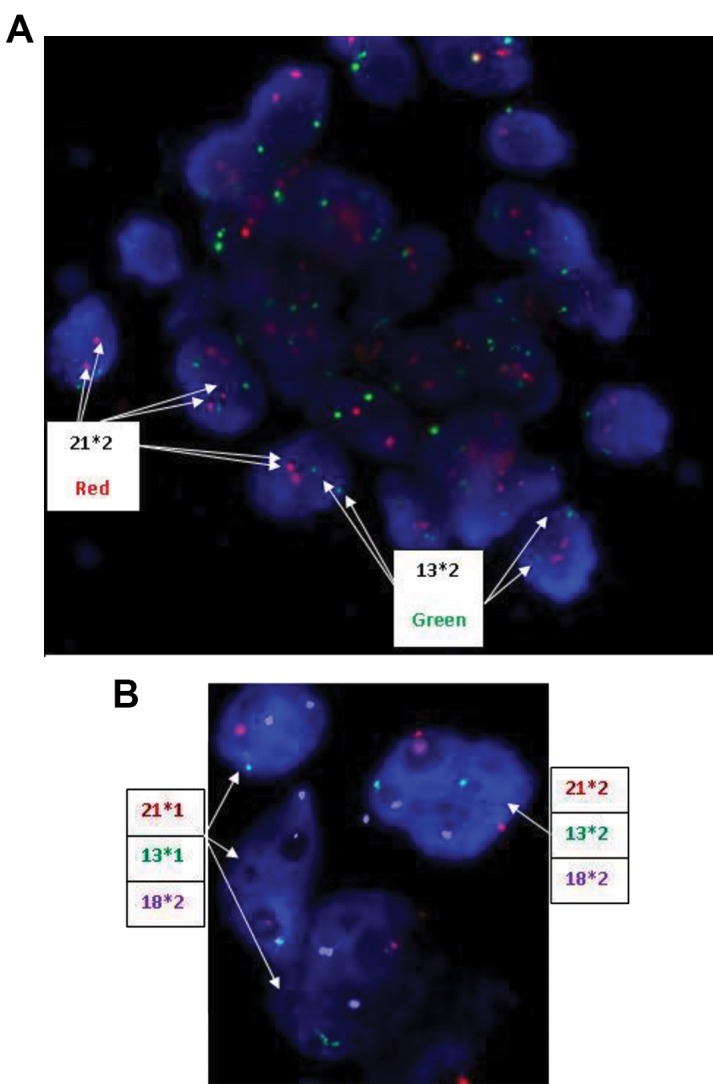
FISH results on blastocyst stage biopsy. **A.** Probe set included 13 (green signal) and 21 (red signal). All of the cells are normal regarding probe 13/21. **B.** Probe set included 13 (green signal), 21 (red signal) and 18 (blue signal). One cell is normal and 3 cells have monosomy 21 and monosomy 13. Overall, the embryo related to B is a mosaic blastocyst.

As presented in Table 2, the aneuploidy prevalence of each chromosome was assessed in total cells of embryos (Fig .4). The blastocysts originated in all groups were similar in total abnormal cells (P=0.179). There was no significant difference between different groups regarding the rate of chaotic genomes (the cells with more than one chromosomal abnormality).

Next, we compared chromosomal abnormalities in the whole blastocysts developed from each group. Our data revealed no significant differences in the abnormality status between twins and control embryos (P=0.845). However, there was a statistically insignificant trend towards a decrease of normal embryos in twins (twin A: 60% and twin B: 57.1%) compared to the controls (71.4%, P>0.05).

**Table 1:** Morphology of the inner cell mass (ICM) and trophectoderm (TE) of blastocysts following *in vitro* splitting


Variables	Control	Twin A	Twin B	P value
	n=21	n= 48	n=42	

ICM (%)				
A	9 (42.9)	2 (4.2)	0	˂0.0001
B	8 (38.1)	18 (37.5)	14 (33.3)	
C	4 (19)	28 (58.3)	28 (66.7)	
TE (%)				˂0.0001
A	16 (76.2)	12 (25)	8 (19)	
B	2 (9.5)	25 (52.1)	19 (45.2)	
C	3 (14.3)	11 (22.9)	15 (35.7)	


The values are presented as the number of embryos (%).

**Table 2:** Aneuploidy prevalence by chromosome


Chromosome	Control	Twin A	Twin B	P value
	n=272	n= 502	n=482	

13	8 (2.9)	9 (1.8)	17 (3.5)	0.237
15	1 (0.4)	3 (0.6)	1 (0.2)	0.621
16	11 (4)	23 (4.6)	26 (5.4)	0.682
18	10 (3.7)	23 (4.6)	24 (5)	0.71
21	5 (1.8)	19 (3.8)	24 (5)	0.097
22	0	4 (0.8)	1 (0.2)	0.17
X	2 (0.7)	5 (1)	0	0.1
Y	0	3 (0.6)	0	0.105
Chaotic cells	1 (0.4)	6 (1.2)	3 (0.6)	0.401
Total abnormal cells	36 (13.2)	83 (16.5)	89 (18.5)	0.179


The values are presented as the number of embryos (%).

## Discussion

Successful experiments in the development of human twin embryos to the blastocyst stage following *in vitro* splitting (6-8) led us to assess their potential for clinical applications. Developmental analyses presented here have proven that human twin embryos were compatible with non-manipulated embryos, as they were similar in their rates of reaching the EB stage. Increasing the number of blastomeres used for the splitting procedure improved the development to all stages. There was no significant difference in the developmental potential between embryos without blastomere disturbance (twin As) and those in which the blastomeres were inputted into the empty ZP one by one (twin Bs). These findings confirmed the theory that the cell-cell interaction between blastomeres is not essential in order to facilitate the development to the blastocyst stage (8).

Morphokinetic assessments revealed no significant difference in the length of time twin embryos took to reach the EBL stage compared to controls. Interestingly, the blastulation time showed a decreasing trend in the twin embryos in group 2 (10-14 blastomeres) compared to the controls. Moreover, A twins reached each stage faster than B twins; however, the differences were not significant. We hypothesized that manipulated blastomeres need extra time for recovering in order to continue the cell cycle. This hypothesis can be supported by some events, especially in recipient embryos during TLM, such as cytoplasmic waves without sign of division, and blastomere displacing and rotation. Furthermore, all embryos in group 2, regardless of being twins or controls, significantly grew faster to the EBL stage. Since the embryos in group 2 had extended culture from pronuclear stage, they needed less time to develop to the blastocyst stage compared to the group 1. These findings demonstrated a similarity in total time needed for blastocyst development for embryos in either of the groups 1 or 2. There was a wide range between the minimum and maximum times of reaching each stage in the twins compared to controls. Twins exhibited a significantly shorter time duration for the compaction (9+ to Mor) and the start of blastulation (Mor to SB) stages than the control embryos. This result is in accordance with findings of Noli et al. (8), suggesting a kind of ‘compensation’ for the lower cell number in twin embryos. In a different assessment, twins from both groups did not differ regarding the duration between the stages.

Based on data from the quality assessment of human twin embryos, splitting resulted in smaller blastocysts with a lower quality of ICM and TE compared to nonmanipulated embryos. A previous study demonstrated that in spite of increasing the number of blastocysts after splitting, the percentage of good quality blastocysts significantly decreased in the mice model (6). In line with our results, Noli and associates had detected a significant difference in size between twins compared to the controls. In addition, they found that the decreased size of blastocysts developed after *in vitro* splitting was due to the decreased number of blastomeres (8). Nevertheless, an animal model study showed offspring of a normal size following *in vitro* splitting because the regulation of cell number occurs after blastocyst formation (19). Mitalipov et al. (20) also found similar ICM:TE and ICM:total cell ratios between twin blastocysts and controls. The presence of NANOG-only positive cells indicates the development potential of the ICM in twins following the splitting procedure (8). The early embryonic blastomeres are totipotent cells and have the individual capacity to develop into both ICM and TE lineages (21). On the other hand, human embryonic genome activation occurs between the 4- to 8-cell stage, when the cells have flexibility (22- 23). So, there is an opinion that the allocation of ICM and TE occurs after embryonic genome activation at the early 8-cell stage before the cells become polarized at both the membrane and cytoplasmic levels. This means that removal of blastomeres after cell polarization does not compromise formation of the ICM. Accordingly, the morning of day 3 was introduced as the best time for blastomere biopsy (24). There are two theories regarding the position of the blastomeres within the embryo and the appearance of two distinct cells lineages i.e. the TE and ICM; The cell polarity model (25) and the inside-outside hypothesis (26). According to these hypotheses, either the outer blastomeres within the embryo or the blastomeres with a perpendicular plane of cleavage participate in the formation of the TE cells (27). Our morphokinetics data showed a derangement in the position of the blastomeres following the *in vitro* splitting procedure. In this method, a smll number of blastomeres were placed into a large space, resulting in an outer position of all blastomeres and subsequently differentiation to TE cells. Our results in regards to the poor quality or lack of ICM in twin blastocysts suggested that lineage determination may take place through the inside-outside model. In a pilot study, we tried *in vitro* splitting in triploid embryos. Next, the developed blastocysts (n=24) from twin embryos were cultured for derivation of human embryonic stem cell (hESC) lines. After three to five days of blastocyst culture, the initial outgrowths of hESC-like cells were generated. After proliferation and passaging, some of the cells expressed hESC and trophoblastic markers, however, no cell line was established (28).

To the best of our knowledge, this is the first study that evaluates the impact of human embryo *in vitro* splitting on chromosomal abnormality and mosaicism. We found that chromosomes 22, 16, 21, and 15 were the main chromosomes involved in cleavage-stage aneuploidies. We also report an improvement in implantation rate with the evaluation of eight critical chromosomes: X, Y, 13, 15, 16, 18, 21, and 22 (29, 30). Our findings in analysing total abnormal cells and embryos showed no significant differences between twin and control embryos. Furthermore, the data showed that the decrease in the number of normal (euploid) twin blastocysts was simultanious with an increase in the proportion of the mosaic blastocysts with no significant differences. Mosaic embryos as a category between normal (euploid) and abnormal (aneuploid) embryos may be lead to a decreased implantation and pregnancy potential as well as an increased risk of genetic abnormalities (31-33). In trisomic mosaicism, it was shown that the embryos with mosaic trisomies of chromosomes 2, 7, 13, 14, 15, 16, 18, and 21 may be in higher risk of developing a child affected with a trisomy syndrome. Therefore, it was advised that the cycles with total mosaic embryos should be canceled until obtaining euploid embryos (31). According to the data on preimplantation genetic screening (PGS), the rate of aneuploidy in the cleavage-stage embryos was 60%, of which approximately 50% were represented by mosaicism where the nature of abnormality was unknown (34-36). There are some reports showing that oocyte manipulation may increase the risk of aneuploidy in subsequent embryos. It was suggested that abberations in cytoskeletal integrity, such as mitochondrial distribution, may reduce the meiotic competence of the oocyte and lead to subsequent mitotic errors at the cleavage-stage and predispose the embryos to chromosomal abnormalities (37). Also, deviances in activity of motor proteins and spindle formation during handling of oocytes are risk factors for non-disjunction and embryo aneuploidy (38). Our results showed that the embryo micromanipulation during *in vitro* splitting does not increase the risk of aneuploidy in the developed blastocysts. It seems micromanipulation in the oocyte may increase the risk of chromosomal abnormality but micromanipulation at the cleavage-stage does not.

Recent studies have introduced comparative genomic hybridization (CGH) and microarray-CGH as more optimal strategies for aneuploidy detection (39), in spite of some of their limitations (40). It is suggested that further studies be conducted with a higher number of donor embryos for *in vitro* splitting, and the use of chromosomal analyses that evaluate whole chromosomal aneuploidies such as CGH-array or next-generation sequencing. In the next step, epigenetic investigations can be performed to rule out the probable effects of *in vitro* splitting on the epigenetic status of the developed blastocysts.

## Conclusion

The current study shows that some developmental timepoints were affected by *in vitro* splitting. This technique increased the number of developed blastocysts and no chromosomal abnormalities were found when compared to controls. However, the developed blastocysts from in vitro splitting were of low quality. This technique may raise the hope to treat poor responders or cases of advanced maternal age in the assisted reproductive technology (ART) program. This study demonstrates that focus on the embryo’s stage at the time of the splitting procedure can improve the outcomes of this technique. These data motivate further attempts of upgrading the *in vitro* splitting program in order to develop more healthy twins.
